# Effect of Hydrogen Inhalation Therapy on Hearing Loss of Patients With Nasopharyngeal Carcinoma After Radiotherapy

**DOI:** 10.3389/fmed.2022.828370

**Published:** 2022-03-31

**Authors:** Xiaofeng Kong, Tianyu Lu, You-Yong Lu, Zhinan Yin, Kecheng Xu

**Affiliations:** ^1^Hydrogen Medicine Institute, The Biomedical Translational Research Institute, Jinan University, Guangzhou, China; ^2^Department of Oncology, Fuda Cancer Hospital, Jinan University, Guangzhou, China; ^3^Key Laboratory of Carcinogenesis and Translational Research (Ministry of Education), Laboratory of Molecular Oncology, Peking University Cancer Hospital & Institute, Beijing, China; ^4^Faculty of Medical Science, The Biomedical Translational Research Institute, Jinan University, Guangzhou, China

**Keywords:** hydrogen inhalation, hydrogen oxygen inhalation, nasopharyngeal carcinoma, radiotherapy, chemotherapy, hearing loss, hearing impairment

## Abstract

**Objective:**

To evaluate the clinical efficacy and safety of hydrogen inhalation in improving hearing loss in patients with long-term survival of nasopharyngeal carcinoma after radiotherapy.

**Methods:**

The eustachian tube dysfunction score, pure tone air conduction threshold, bone conduction threshold, the score of tympanogram and otoscope were prospectively observed in patients with deafness after radiotherapy only or combined radiotherapy and chemotherapy for nasopharyngeal carcinoma. Paired t test and one-way analysis of variance were used to analyze the data before and after treatment.

**Results:**

A total of 17 patients were observed. The median time from radiotherapy to now was 228 months, and the median time from the diagnose of deafness to now was 92 months. After 4 weeks of hydrogen inhalation, the score of eustachian tube dysfunction, air conduction and bone conduction hearing thresholds were significantly reduced, *P* values were 0.0293, 0.0027, 0.0404, respectively. The mean air-bone gap, the score of otoendoscopy and tympanogram were also decreased, but the differences were not significant (*P* = 0.2079, *P* = 0.0536, *P* = 0.1056). Patients with radiotherapy alone and concurrent chemo-radiotherapy had significantly lower air conduction hearing threshold after hydrogen absorption (*P* = 0.0142, *P* = 0.0495). The results of air and bone hearing thresholds before, 4 and 12 weeks after hydrogen inhalation showed a descending trend. The air and bone hearing thresholds before hydrogen inhalation were 74.69 ± 27.03 dB and 45.70 ± 21.58 dB, respectively. At the 12th week, the mean values of air and bone hearing thresholds were the lowest, which were 66.88 ± 20.88 dB and 40.94 ± 18.93 dB, respectively, but there was no significant difference in air and bone hearing thresholds among all groups (*P* = 0.6755, *P* = 0.7712). After hydrogen inhalation treatment, no adverse reactions such as nosebleed, chest pain, dyspnea, nausea, vomiting, dizziness, earache and allergic reaction were observed.

**Conclusion:**

This is the first prospective study on the effect of hydrogen inhalation on hearing improvement in patients with deafness after radiotherapy/chemotherapy for nasopharyngeal carcinoma, suggesting that continuous hydrogen inhalation may be an alternative rehabilitation therapy for these patients.

## Introduction

As one of the most common malignant tumors in the head and neck, nasopharyngeal carcinoma (NPC) is primarily treated with radiotherapy alone or radiotherapy combined with chemotherapy. Patients' 5-year survival rate can be 77.9–87.4% (1–4). Due to the fact that the entire auditory system including the middle ear, cochlea, auditory nerves, brainstem and auditory cortex are all located at or close to the irradiation area for nasopharyngeal cancer treatment, the radiation from radiotherapy inevitably implicates the aforementioned anatomical structure of the nasopharynx. Radiotherapy is usually followed by such complications as secretory otitis media, ossicular chain necrosis, and cochlea & auditory nerve damage. Hearing impairment is the most common late-stage adverse reaction after nasopharyngeal carcinoma radiotherapy (5, 6). As patients survive longer, this complication becomes progressively worse and seriously compromises the quality of life. So far, there has been no special treatment, making tumor rehabilitation a major challenge (7, 8).

Hydrogen (H_2_) is the lightest and smallest gas in nature. Dole et al. (9) first reported the therapeutic effect of high-pressure and high-concentration hydrogen on mouse models with squamous cell carcinoma in 1975. In 2001, Gharib et al. (10) proved that inhalation of high-pressure hydrogen alleviates the inflammatory response caused by liver parasite infection. In 2007, Ohsawa et al. (11) found that low-concentration hydrogen selectively neutralizes hydroxyl radicals (OH) and peroxynitrite (ONOO-); and possesses antioxidant properties. In recent years, a large number of preclinical studies and a few clinical studies have observed the preventive and therapeutic effects of this gas on different system diseases (12), including the effects on inhibiting cancer cell growth, invasion and metastasis; and in reducing adverse reactions of radiotherapy/chemotherapy (12–15). In respect of the hearing system, some studies have revealed that molecular hydrogen protects hair cells of the hearing system through antioxidant effects, and ameliorates hearing (16–19).

This study observes the changes of hearing-related indexes before and after hydrogen inhalation by 17 patients with 5-year-long or longer hearing impairment after nasopharyngeal carcinoma treatment, with a focus on assessing the effectiveness and safety of hydrogen in improving hearing. As far as we know, this is the first report of research findings in this domain. With the hope to reduce hearing loss after radiotherapy or chemotherapy, we wish to provide a new, safe and effective treatment and rehabilitation option for patients with nasopharyngeal carcinoma.

## Subjects and Methods

### Subjects

From May 2019 to October 2020, a total of 34 nasopharyngeal carcinoma patients with post-radiotherapy hearing loss from the Nasopharyngeal Carcinoma Group of the Light of Life Cancer Rehabilitation Association of Guangdong Province were enrolled in this study after signing informed consent. According to the registered clinical trial (ClinicalTrials.gov, ID: NCT03818347; Registration Date: January 24, 2019), all the enrolled patients met the following conditions: (1) Diagnosed with Stage I to IVb nasopharyngeal carcinoma after pathological examinations; (2) Received conventional radiotherapy (radiotherapy) or radiotherapy combined with chemotherapy (chemoradiotherapy) 5 years ago; (3) Hearing and hearing test results before anti-tumor treatment were normal; (4) Experienced hearing loss at the age of 60 or lower, which excluded the probability of presbycusis; (5) Having inhaled hydrogen for at least 4 weeks. Patients with the following conditions were excluded from this study: (1) Severe hypertension, diabetic autoimmune disease, history of head trauma, and experience of long-time working in noisy environment; (2) Recurrence or metastasis of existing tumor(s); (3) Middle ear disease or hearing impairment developed before anti-tumor treatment; (4) Head and neck radiotherapy due to other reasons; (5) Middle ear effusion as evidenced by otoscopy.

The three patients who did not keep inhaling hydrogen daily and 14 patients who failed to complete regular relevant examinations were excluded from analysis. A total of 17 patients (34 ears) completed the study eventually.

The 17 patients, including seven males and 10 females, aged from 47 to 67 years old, with a median of 58 years old. According to AJCC (American Joint Committee on Cancer) tumor staging (7th edition), 10 cases were in Stage I, 4 cases in Stage II, 1 case in Stage III, and 2 cases in Stage IV. Eleven of the cases had received radical radiotherapy alone; and 6 cases radiotherapy combined with chemotherapy. During radiotherapy, the radiation dose of 68–72 Gy was applied to nasopharyngeal carcinoma and draining lymph node areas; 70.0 Gy to positive cervical lymph nodes; and 50.0–60.0 Gy to negative cervical lymph nodes. The patients were exposed to radiation 34 to 38 times in total once a day for five times a week, with dose fractionation set at 1.8–2.0 Gy each time. Concurrent chemotherapy was performed with the regimen 40 mg/m2 cisplatin (DDP) given through intravenous drips once a week. It has been 100 to 355 months (a median of 259 months) since the aforementioned treatment was completed. Hearing loss occurred 24 to 289 months after the completion of radiotherapy or chemoradiotherapy, with a median of 117 months. It persisted for 30–186 months, with a median of 90 months (Table 1). The patients received hearing loss treatments including: glucocorticoid treatment in 12 cases, acupuncture in seven cases, Chinese medicine treatment in 16 cases, and hyperbaric oxygen treatment in one case. None of the patients showed improvement from the treatments.

**Table 1:** Basic information of the 17 patients with hearing loss after nasopharyngeal carcinoma radiotherapy/chemoradiotherapy.

**Case no**.	**Age (Y)**	**Gender**	**Staging at time of treatment**	**Tumor treatment**	**End of treatment till now (month)**	**End of treatment to discovery of hearing loss (month)**	**End of treatment to receiving hydrogen treatment (month)**	**Hydrogen inhalation duration (week)**
1	66	Female	I	Radiotherapy	300	246	54	12
2	47	Male	III	Chemoradiotherapy	173	83	90	12
3	63	Female	I	Chemoradiotherapy	220	114	106	12
4	62	Male	II	Radiotherapy	271	115	156	8
5	47	Female	I	Radiotherapy	120	54	66	12
6	67	Female	I	Radiotherapy	147	117	30	8
7	64	Female	IV	Chemoradiotherapy	259	114	145	12
8	63	Female	II	Chemoradiotherapy	113	54	59	12
9	60	Male	II	Radiotherapy	275	149	126	4
10	53	Male	I	Radiotherapy	354	168	186	8
11	53	Female	I	Radiotherapy	355	289	66	4
12	60	Female	II	Chemoradiotherapy	183	117	66	8
13	56	Male	I	Radiotherapy	268	130	138	4
14	67	Female	I	Radiotherapy	310	196	114	8
15	48	Female	IV	Chemoradiotherapy	100	56	44	12
16	54	Male	I	Radiotherapy	114	24	90	12
17	60	Male	I	Radiotherapy	228	144	84	8

### Hydrogen Inhalation Method

The subjects inhaled hydrogen-oxygen mixture, which contained 2.0 L/min hydrogen and 1.0 L/min oxygen (produced by hydrogen-oxygen nebulizer AMS-H-03, Shanghai Asclepius Meditec Co., Ltd.), through nasal tubes in quite conditions. Daily inhalation lasted 3–6 h for 4–12 weeks, with eight cases for 12 weeks, six cases for 8 weeks and three cases for 4 weeks. The test period was divided into three periods for assessment: before hydrogen treatment, after 4 weeks of hydrogen treatment, and after 12 weeks of hydrogen treatment.

### Eustachian Tube Dysfunction Questionnaire-7 (ETDQ-7) Survey

Eustachian tube dysfunction questionnaire-7 (ETDQ-7) survey (20, 21) was adopted. The scale included seven items, representing seven levels (from 1 to 7 scores) according to the severity of symptoms. The higher the score, the more severe the symptoms. Patients were given face-to-face questionnaire surveys 1–3 days before hydrogen inhalation and 1–3 days after 4 weeks of hydrogen inhalation.

### Tympanic Membrane Detection

Scoring was performed according to otoscope-based examination: 1 score: tympanic membrane turbidity and thickening; 2 scores: tympanic membrane congestion and indentation as well as disappearance of light cone; 3 scores: tympanic membrane effusion; and 4 scores: tympanic membrane perforation.

### Tympanogram Changes

Acoustic impedance tester was used to assess the changes in calm breathing tympanogram (17, 18). Tympanogram scoring rules were: 3 scores for type Ad; 2 scores for type A or As; 1 score for type C; and 0 score for type B.

### Pure-Tone Hearing Threshold Testing

Pure-tone hearing threshold testing (with AT235 from a Danish international hearing health care company) was carried out in a standard sound-proof chamber. With acoustic standard set at GB/T16296 and reverberation time at 0.3 ± 0.15 s, pure-tone hearing thresholds in conditions of 0.5, 1, 2, 4, and 8 kHz were tested in sequence. The average air-conduction threshold, bone-conduction threshold, and air-bone gap under 0.5–4 kHz were calculated. The average pure tone hearing threshold (PTA) of four frequencies divides hearing loss into the following levels: normal hearing is defined as the mean of pure tone hearing threshold ≤ 25 dB; 26–40 dB HL was mild hearing loss, 41–55 dB HL was moderate hearing loss, 56–70 dB HL was moderately severe hearing loss, 71–90 dB HL was severe hearing loss, and ≥91 dB HL was very severe hearing loss. Pure tone hearing threshold test by hearing detection professional testing.

### Safety Assessment

According to Common Terminology Criteria for Adverse Events (CTCAE-4.0 version) defined by the National Cancer Institute of U.S. Department of Health and Human Services (22), symptoms or side effects are graded from Grade 1–5. Safety evaluation was conducted once every 4 weeks after hydrogen inhalation, covering symptoms of nose bleeding, cough, chest pain, dyspnea, nausea, vomiting, dizziness, earache and allergic reaction.

### Statistical Analysis

Measurement data were expressed as x ± s. The data before and after hydrogen inhalation were compared by paired *t* test. Different hydrogen uptake durations were compared using one-way analysis of variance. P ≤ 0.05 indicated that the difference is statistically significant. All analyses and figures were produced using GraphPad Prism 5.0 (GraphPad software, San Diego, CA, USA).

## Results

### Eustachian Tube Function

A total of 17 cases received the survey using 7-item questionnaires about eustachian tube dysfunction. 34 questionnaires were returned back, with 100% effective response rate. The score before hydrogen inhalation was 3.25 ± 2.00, and that after 4 weeks of hydrogen inhalation was 2.59 ± 1.65. The difference between the two groups was statistically significant (*P* < 0.05) (Table 2).

**Table 2:** Eustachian tube dysfunction 7-item score, otoscopy score and tympanogram score.

	**Number of ears**	**Before hydrogen- oxygen inhalation x ±s**	**4 weeks after hydrogen- oxygen inhalation x ±s**	***P* value**
Eustachian tube disfunction 7-item score		3.25 ± 2.00	2.59 ± 1.65	0.0293^*^
Otoscopy score	34	1.76 ± 1.26	1.44 ± 1.24	0.0536
Tympanogrom score	34	0.68 ± 0.98	0.97 ± 1.06	0.1056

* *P < 0.05*.

### Scoring of Tympanic Membrane Under Otoscope and Tympanogram

Tympanic membrane perforation occurred in five ears before hydrogen inhalation and persisted after treatment. After 4 weeks of hydrogen inhalation, the tympanic membrane score based on otoscopy stabilized in 10 cases, improved in 6 cases, and worsened in one case. However, the difference compared to before hydrogen inhalation was not statistically significant (*P* > 0.05). Tympanogram scores before and after hydrogen inhalation showed no statistically significant difference (*P* > 0.05) (Table 2).

### Hearing Changes

#### Hearing Changes After 4 Weeks of Hydrogen Inhalation

**Figure 1:**
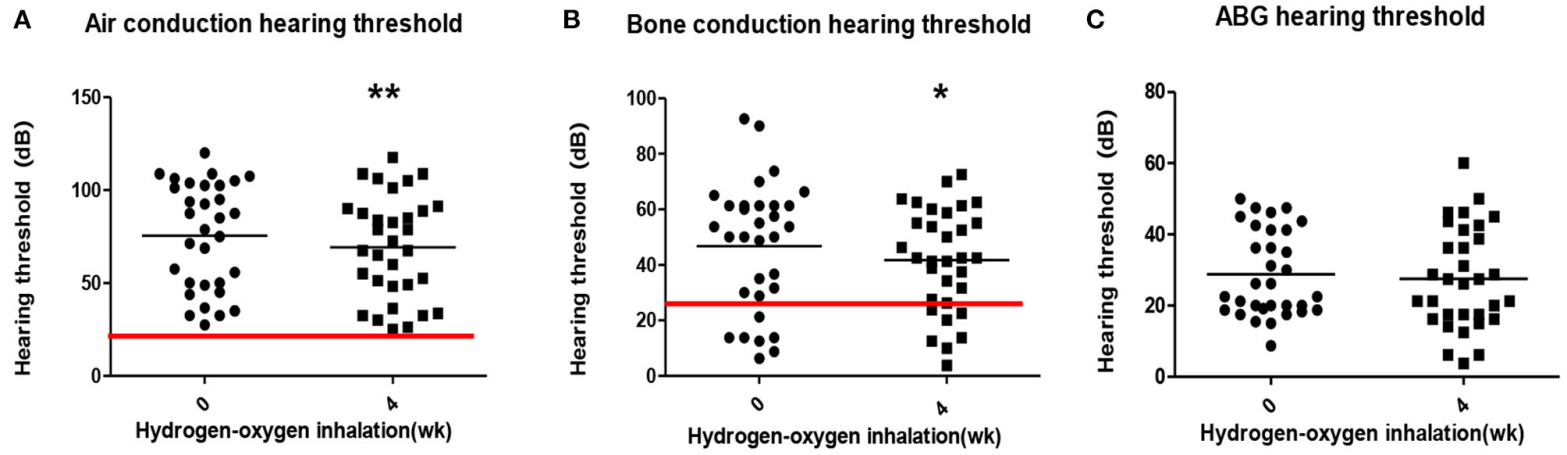
Pure tone hearing threshold tests result before and after hydrogen-oxygen mixed gas inhalation on hearing loss patients with long-term survival after combined modality treatment of nasopharyngeal carcinoma. **(A)** Test results of the air conduction hearing threshold. **(B)** Test results of the bone conduction hearing threshold. **(C)** Test results of the air bone gap (ABG) hearing threshold. The parallel red long lines in the figure represent the normal range,the black short lines represent the average value at each time point. Data are analyzed by paired *t* test. ^*^P < 0.05, ^**^P < 0.01.

The average air conduction, bone conduction and air-bone gap before hydrogen inhalation in 17 patients (34 ears) were 77.46 ± 28.12, 46.76 ± 20.73, and 30.70 ± 11.12 dB, respectively. After 4 weeks of hydrogen inhalation treatment, both air conduction threshold and bone conduction threshold were significantly lower than those before hydrogen inhalation, being 73.35 ± 28.20 dB (*P* = 0.0027) and 44.56 ± 19.50dB (*P* = 0.0404), respectively (Figures 1A,B). Among them, the threshold of air conductivity was significantly improved, with a decrease in 64.71% of the patients (Table 3). The mean air bone gap(ABG) decreased from that before treatment (28.78 ± 13.44 dB), but the difference was not statistically significant (*P* = 0.2079) (Figure 1C).

**Table 3:** Air conductance threshold improvement before and 4 weeks after hydrogen and oxygen inhalation therapy.

**Air-conduction hearing threshold improvement decibel (dB)**	**Number of ears**	**Percentage (%)**
≤ 0	12	35.29%
0–10	18	52.94%
11–20	3	8.82%
21–30	1	2.94%

#### Hearing Improvement in Patients With Different Initial Treatment Regimens for Tumors

**Figure 2:**
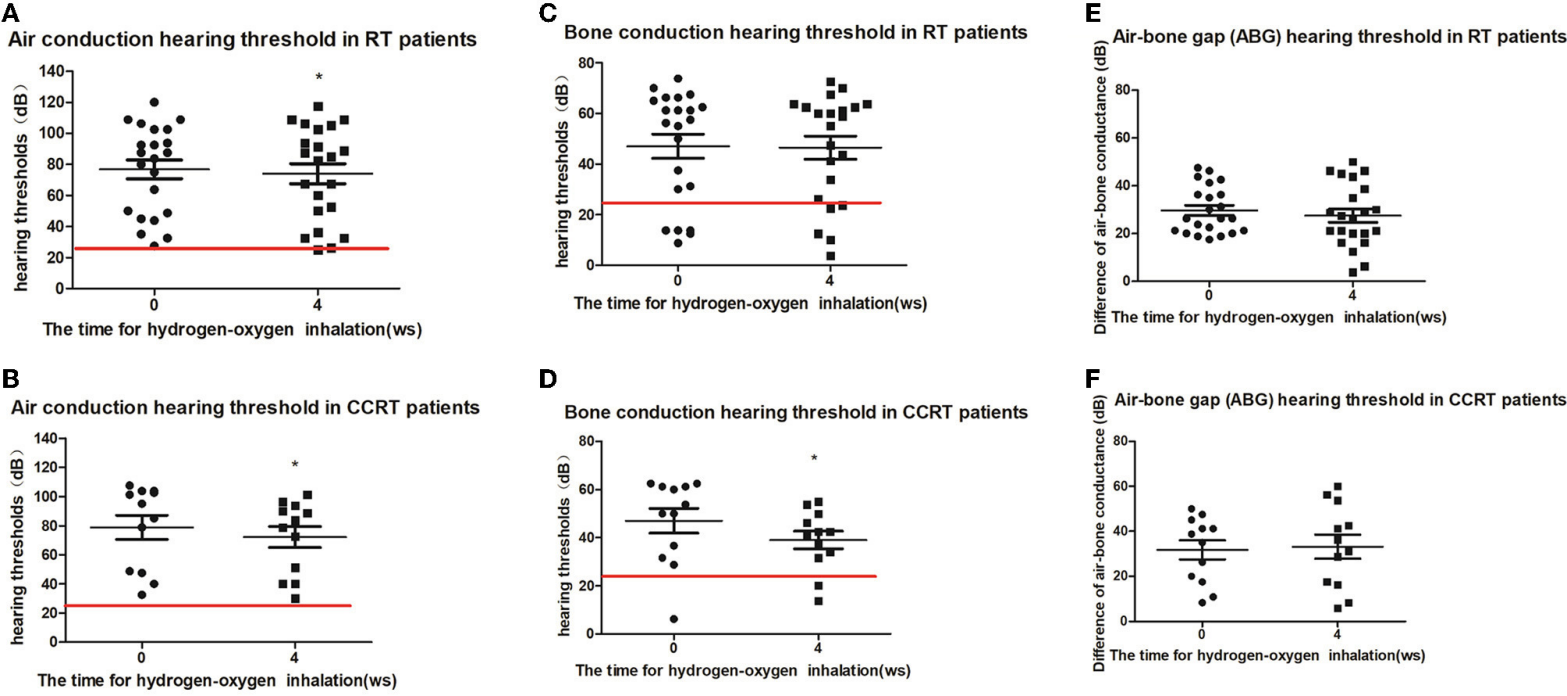
Pure tone hearing threshold tests result before and after hydrogen-oxygen mixed gas inhalation on hearing loss patients with long-term survival after radiotherapy only and concurrent chemoradiotherapy of nasopharyngeal carcinoma. **(A)** Test results of the air conduction hearing threshold in RT patients. **(B)** Test results of the air conduction hearing threshold in CCRT patients. **(C)** Test results of the bone conduction hearing threshold in RT patients. **(D)** Test results of the bone conduction hearing threshold in CCRT patients. **(E)** Test results of the air bone gap (ABG) hearing threshold in RT patients. **(F)** Test results of the air bone gap(ABG) hearing threshold in CCRT patients. The parallel red long lines rep in the figure represent the normal range. Data are analyzed by paired t test. ^*^P < 0.05.

According to the initial methods of tumor treatment, 17 patients (34 ears) were separated to radiotherapy group (22 ears) and concurrent chemoradiotherapy group (12 ears). The average air-conduction threshold, bone-conduction threshold and air-bone gap of the radiotherapy group before hydrogen inhalation were 76.70 ± 28.25, 47.05 ± 22.30, and 29.66 ± 9.86 dB, respectively. After 4 weeks of hydrogen inhalation, the air-conduction threshold showed decline with significant difference (73.98 ± 30.29 dB, *P* = 0.0142, Figure 2A); and bone-conduction and air-bone gap hearing thresholds showed decrease without significant difference (*P* = 0.6137 and *P* = 0.1699, respectively. Figures 2C,E). Before hydrogen inhalation, the average air-conduction threshold, bone-conduction threshold and air-bone gap of patients with concurrent chemoradiotherapy were 78.85 ± 28.55, 47.05 ± 17.72, and 31.81 ± 14.63 dB, respectively. After hydrogen inhalation, the thresholds of air conduction and bone conduction were significantly reduced, being 72.19 ± 25.13 dB and 39.04 ± 12.61 dB, respectively. *P* values were 0.0495 and 0.0134, respectively (Figures 2B,D). The average air-bone gap between the two groups was increased (33.15 ± 18.48 dB) compared to before treatment, but the difference was not statistically significant (*P* = 0.7283, Figure 2F).

#### Hearing Threshold Changes in Patients With Different Hydrogen Inhalation Durations

**Figure 3:**
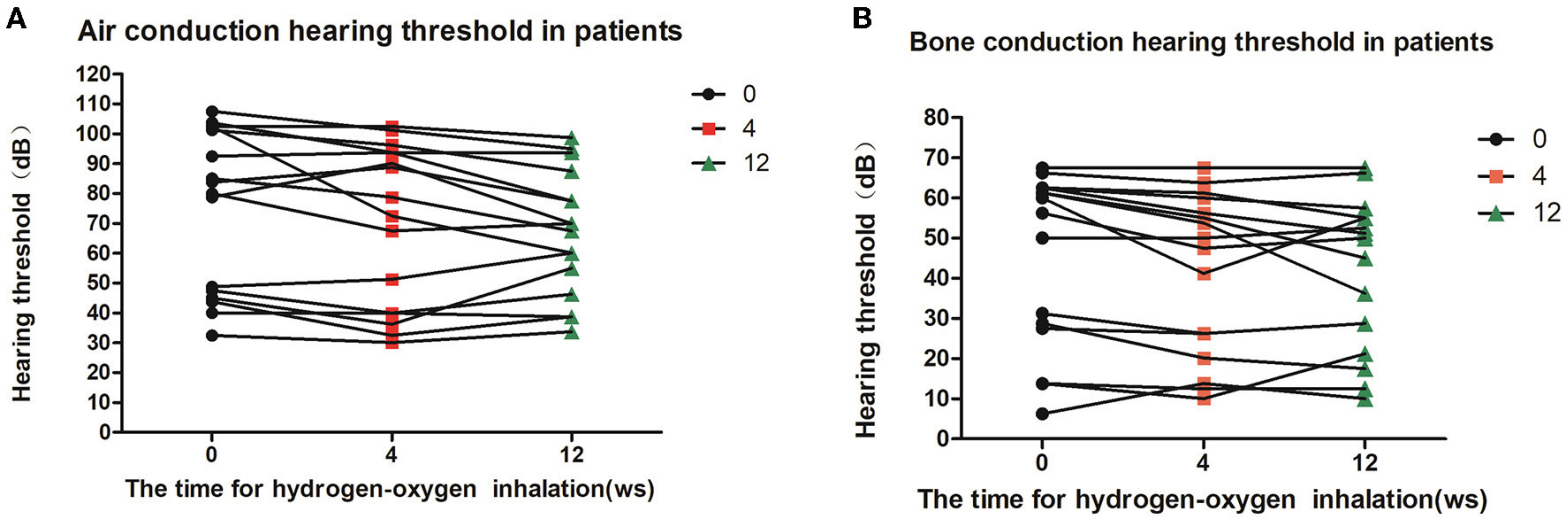
Patients' hearing changes at different time points of hydrogen-oxygen inhalation. **(A)** Diagram of changes of air conduction hearing threshold at different time point after hydrogen-oxygen gas inhalation; **(B)** Diagram of changes of bone conduction hearing threshold at different time point after hydrogen-oxygen gas inhalation. One-way analysis of variance was used for data analysis.

Eight of the deaf patients (16 ears) were treated with hydrogen inhalation for 12 weeks. The results of air conduction threshold and bone conduction threshold were compared before, 4 weeks and 12 weeks after hydrogen inhalation treatment, showing a downward trend. Before hydrogen inhalation, air and bone hearing thresholds were 74.69 ± 27.03 dB and 45.70 ± 21.58 dB, respectively. At 12 weeks of treatment, the mean values of air conduction threshold and bone conduction threshold were the lowest, which were 66.88 ± 20.88 dB and 40.94 ± 18.93 dB, respectively. However, there was no significant difference in air and bone hearing threshold among all groups (*P* = 0.6755,*P* = 0.7712, Figures 3A,B).

#### Patients' Hearing Changes After Suspension of Hydrogen-Oxygen Inhalation

**Figure 4:**
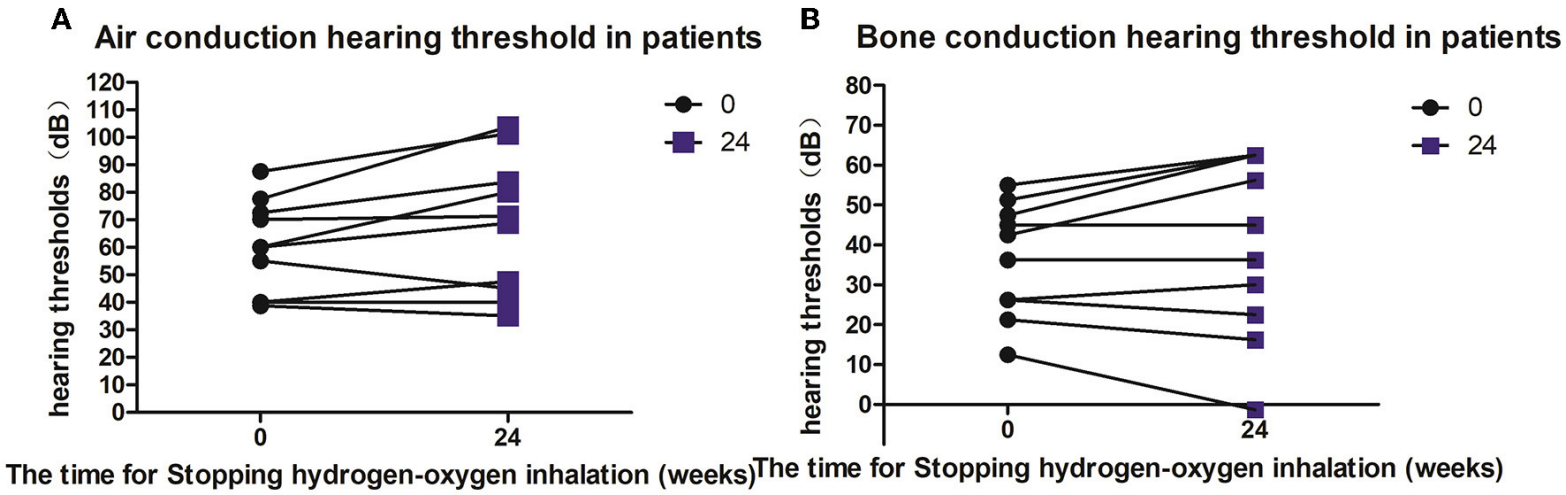
Hearing changes in patients after hydrogen and oxygen inhalation therapy was discontinued. **(A)** Diagram of changes in air conduction hearing threshold after stopping hydrogen-oxygen inhalation therapy for 24 weeks; **(B)** Figure of changes in bone conduction hearing threshold after stopping hydrogen-oxygen inhalation therapy for 24 weeks. Paired *t* test was used for data analysis.

Five patients (10 ears) were given hearing checkups 6–9 months after they stopped hydrogen-oxygen gas inhalation. According to the checkup results, the bone- and air-conduction hearing thresholds of three ears were stable or showed continuous improvement; and the hearing thresholds of seven ears increased in decibels. Average decibels of both air- and bone-conduction hearing thresholds for the affected ears were higher than before, but no significant difference was observed with *P* value of 0.0596 and 0.3473 respectively (see Figures 4A,B).

### Adverse Reactions Related to Hydrogen-Oxygen Inhalation

No such common adverse reaction as nose bleeding, chest pain, dyspnea, nausea, vomiting, dizziness, earache or skin allergy was observed after hydrogen-oxygen inhalation. Ten patients (58.8%) complained of mild cough before hydrogen inhalation, and 11 patients (64.7%) complained of cough 4 weeks after hydrogen inhalation. Earache occurred before hydrogen inhalation in one of the cases and no change of earache after the treatment. As time passed, the aforementioned symptoms did not worsen (Table 4).

**Table 4:** Adverse reactions at different time points after hydrogen inhalation.

	**Before hydrogen inhalation (17 patients)**					**4 weeks after hydrogen inhalation(17 patients)**					**8 weeks after hydrogen inhalation (8 patients)**					**12 weeks after hydrogen inhalation (8 patients)**				
Adverse reaction	1	2	3	4	5	1	2	3	4	5	1	2	3	4	5	1	2	3	4	5
Nose bleeding	0	0	0	0	0	0	0	0	0	0	0	0	0	0	0	0	0	0	0	0
Cough	10 (58.82%)	0	0	-	-	11 (64.70%)	0	0	-	-	7(87.5%)	0	0	-	-	6(75%)	0	0	-	-
Chest pain	0	0	0	-	-	0	0	0	-	-	0	0	0	-	-	0	0	0	-	-
Dyspnea	0	0	0	0	0	0	0	0	0	0	0	0	0	0	0	0	0	0	0	0
Nausea	0	0	0	-	-	0	0	0	-	-	0	0	0	-	-	0	0	0	-	-
Vomiting	0	0	0	0	0	0	0	0	0	0	0	0	0	0	0	0	0	0	0	0
Dizziness	0	0	0	-	-	0	0	0	-	-	0	0	0	-	-	0	0	0	-	-
Earache	1(5.88%)	0	0	-	-	1(5.88%)	0	0	-	-	1(12.5%)	0	0	-	-	1(12.5%)	0	0	-	-
Allergic reaction	-	-	0	0	0	-	-	0	0	0	-	-	0	0	0	-	-	0	0	0

## Discussion

### Hearing Impairment After Radiotherapy

Nasopharyngeal carcinoma (NPC) is the most common malignant tumor of the head and neck, with more than half of the world's cases found in China. And such cases are mostly seen in southern and southeastern coastal areas of China. Currently, radiotherapy is the most effective treatment for NPC. As radiotherapy technology advances, the efficacy has improved gradually. Early-stage NPC patients'5-year survival rate may reach as high as 95% (23). As the most common adverse reaction after radiotherapy, hearing impairment takes place in the following three circumstances: (1) Hearing impairment occurs directly after radiotherapy and is usually temporary. Patients recover from it after several weeks or months (24). (2) Sudden deafness or sudden hearing loss occurs without any apparent cause at a certain time point after the end of radiotherapy (25). (3) Hearing loss occurs gradually after radiotherapy or chemoradiotherapy after a “latency period” of varying lengths (26). This is the case with the group of patients included in this study. The median interval (shortest at 100 months) from the completion of radiotherapy or chemoradiotherapy till now is 259 months. Hearing loss occurred at the median time of 117 months (ranging from 24 months to 285 month) after the completion of radiotherapy or chemoradiotherapy. It persisted for a median time of 90 months (shortest at 30 months). In these cases, there is neither evidence of the existence or recurrence of any primary tumor, nor other factor that could have caused hearing impairment.

Hearing loss is a major late complication for long-term surviving patients after radiotherapy or radiotherapy combined with chemotherapy. As a patient survives longer, the incidence of hearing loss increases, and hearing loss becomes more serious (27). The hearing loss rate of patients who survive more than 5 years can reach 60.5–94% (6, 28–30). Due to the lack of targeted treatments, existing treatments are usually based on symptomatic treatments, with limited effects (31). It has been reported that hearing improved after sudden deafness was treated with Intratympanic steroids (32). All the patients in this study group received a variety of treatments, including oral steroids or ear drops, acupuncture and hyperbaric oxygen. But the treatments failed to relieve hearing damage or prevent the aggravation.

### Observation on Hydrogen-Based Treatment of Hearing Loss

In 2020, we (33) reported about three cases of NPC patients with hearing loss after radiotherapy. The patients had significant improvement in hearing after hydrogen inhalation. In a real-world evidence survey of cancer patients who took hydrogen inhalation voluntarily as a means of rehabilitation, a 63-year-old male patient with nasopharyngeal carcinoma radiotherapy 19 years ago was observed. After radiotherapy, the patient experienced hearing loss, which showed no substantial improvement in spite of symptomatic treatments, and had to use hearing aid for smooth communication. On January 17, 2019, he started to inhale hydrogen on a daily basis. After March 2019, his hearing improved tremendously, ridding him of the hearing aid. The patient's experience led us to the unexpected discovery that hydrogen might be effective in treating hearing loss. Therefore, with a request and support from the Nasopharyngeal Cancer Group of Light of Life Cancer Rehabilitation Association in the patient's place of living, we conducted this prospective study. The 17 patients in the study had clear history of nasopharyngeal carcinoma and radiotherapy or chemoradiotherapy. In quiet environment, the patients inhaled hydrogen (mixture of hydrogen and oxygen) through nasal tubes at least 3 h a day for more than 4 weeks. Results indicated that hearing was improved in most cases. After 4 weeks of hydrogen inhalation, the patients' eustachian tube function scale scores were significantly lower than before treatment; and both their air- and bone-conduction hearing thresholds of the affected ears declined notably. As the treatment continued, the patients' hearing decibels of the affected ears decreased more than before. After hydrogen inhalation was stopped, hearing in some of the cases continued to improve. For subgroups with different initial treatments, the air-conduction hearing thresholds of ears affected by radiotherapy alone and air- and bone-conduction hearing thresholds of ears affected by concurrent radiotherapy and chemotherapy were significantly improved.

It is worth noting that the hydrogen inhalation is very safe. Few patients in the study experienced hydrogen-specific adverse reactions.

Hydrogen inhalation by the 17 patients in this study group was carried out under strict medical supervision and was not given any other medical intervention during the entire period. Therefore, it can be concluded that the hearing improvement found in these patients is undoubtedly attributable to hydrogen inhalation.

### Mechanism of Hydrogen in Improving Hearing Loss

The mechanism by which hydrogen improves hearing loss in patients with nasopharyngeal carcinoma after radiotherapy or chemoradiotherapy remains unclear. However, if we connect the biological effect of radiation (or chemotherapy drugs) with the mechanism of action of hydrogen, it is not difficult to note that hydrogen represents a “tit-to-tat” “pathogenesis treatment” for post-radiation hearing loss.

First of all, hydrogen has anti-oxidant and anti-inflammatory effects that resist oxidative stress and inflammation caused by radiotherapy. In the process of radiotherapy, reactive oxygen species (ROS), especially hydroxyl radicals (OH-) and peroxy-nitrosamine, are generated in large volume. Excessive levels of ROS destroy the composition of mitochondrial electron transport chains; trigger imbalance of intracellular redox systems (34); and cause lipid peroxidation, protein misfolding and DNA strand breakage. In the meantime, they activate the JNK signaling system; up-regulate pro-oxidant genes; and inhibit antioxidants related to nuclear factor-e2 related factor (Nrf2) (35), thereby causing oxidative stress and meanwhile inducing matrix metalloproteinases (MMP); triggering secretion of inflammatory chemokines including tumor necrosis factors (TNFs), interleukin (IL)−1, IL-6 and IL-8; and precipitating inflammation. Peroxidation and inflammation promote cell apoptosis. Subsequently, pro-fibrotic cytokines such as platelet-derived growth factors (PDGFs), insulin-like growth factors (IGFs) and basic fibroblast growth factors (FGFs) are released, promoting differentiation of monocytes into M2 macrophages; strengthening fibroblasts to proliferate and differentiate into myofibroblasts; and then amplifying inflammation and fibrosis formation. Radiation can also induce the expression of several miRNAs including miRNA-1 and miRNA-21. And the up-regulation of miRNA-21 expression is related to fibrosis.

The dose of radical radiotherapy for nasopharyngeal carcinoma is far higher than the normal tissue tolerance dose. Therefore, oxidative stress, inflammation and fibrosis that accompany radiotherapy inevitably damage the auditory system in the radiation field (36–39), leading to middle ear, inner ear and auditory nerve cell damages. Eventually, conductive, sensorineural or mixed hearing loss occurs. Although intensity-modulated radiotherapy helps reduce the dose to surrounding normal tissues (6, 40, 41), ear symptoms are still unavoidable for nasopharyngeal cancer survivors (42–44). The hearing loss of patients receiving radiotherapy combined with chemotherapy is usually more severe than that of patients with radiotherapy alone (39). This might be attributed to the fact that cisplatin, which is commonly used in chemotherapy, has a strong effect on inducing ROS generation.

Hydrogen molecule is a weak reducing agent with very low molecular weight. It can quickly diffuse and pass through cell membranes and lipid bilayers; reach cell nuclei and mitochondria where abundant invasive ROS gather; and selectively neutralize highly reactive toxic ROS (such as ∙OH) directly (11). Further studies have revealed that hydrogen regulates the Nrf2 pathway (45). Nrf2 is considered to be an important regulator of electrophilic/antioxidant homeostasis, and is especially capable of maintaining the functional integrity of cells under oxidative stress conditions. Hydrogen helps activate the Nrf2-Keap1 system; induce activation of antioxidant response elements (AREs); and promote expression of multiple cytoprotective proteins such as glutathione, catalase (CAT), superoxide dismutase (SOD), glutathione peroxidase and heme-1 oxygenase; activate transcription factor FoxO1; reduce damage of OH to mitochondria; and inhibit overproduction of ROS. Moreover, hydrogen inhibits infiltration of phagocytes to sites of inflammation and subsequent release of reactive substances; and down-regulates various pro-inflammatory and inflammatory cytokines including interleukin (IL)-1β, IL-6, TNF-α and intracellular adhesion molecules (ICAM)-1, thereby achieving anti-inflammatory effect (46, 47). Hydrogen also weakens abnormal expression of miRNA induced by radiation and reduces fibrotic damage (45).

According to the findings of existing studies, hydrogen treatment helps significantly mitigate hearing loss caused by cisplatin; reduce Organ of Corti hair cell damage from cisplatin; improve levels of malondialdehyde (MDA) and isoprostanes F2α (8-iso-PGF2α) in serum and cochlea tissues (48); significantly increase the number of residual acoustic hair cells in the cochlea; and reduce formation of hydroxyl radicals in the cochlea (49). These facts support the aforementioned mechanism.

Second, hydrogen has cytoprotective activity that improves cell apoptosis induced by radiotherapy. Apoptosis plays an important role in progression of radiation injury. Hydrogen significantly inhibits ectopic expression of death promoter Bcl-2 related X protein (bax) and expression of caspase-3, and meanwhile, promotes expression of the anti-apoptotic protein Bcl-2, thereby achieving cytoprotective activity (50, 51).

Third, hydrogen improves blood perfusion and alleviates vascular damage as a result of radiotherapy. Vascular injury and endothelial dysfunction play key roles in development of radiation injury (52). Within a few minutes of exposure to ionization radiation (IR) and as ROS is generated in excessive amount, vascular protectant nitric oxide (NO) is eliminated, triggering nitrosylation of protein tyrosine residues and lipid peroxidation. Ultimately, vasomotor response is weakened, and vascular stenosis appears (53, 54). After radiation, NADPH oxidases (NOXs), especially NOX2 and NOX4 that are abundantly expressed in vascular endothelial cells, are up-regulated. This promotes excessive production of ROS; changes calcium homeostasis and calcium metabolism disorders; and triggers antifibrinolysis-coagulation cascade action, leading to blood clotting and vascular occlusion (55–57). Radiation facilitates migration of monocytes to inner membranes, induces expression of inflammatory adhesion molecules, enhances adhesive capacity of monocytes, recruits monocyte chemotactic protein-1 into inner membranes, absorbs low-density lipoproteins, and promotes arteriosclerosis (58), which eventually leads to arterial stenosis and lower blood perfusion. Since the neck is usually included in the irradiation scope of radiotherapy for patients with nasopharyngeal cancer, carotid artery damage (59) and cochlear vascular damage are inevitable, causing blood supply disorders. This may also directly incur damage to outer hair cells and spiral ganglion cells of the cochlea (60).

Evidence suggests that hydrogen protects damaged blood vessels and improves blood perfusion, including inhibiting degradation of cyclic guanosine monophosphate (cGMP) through phosphodiesterase, increasing cGMP levels and promoting protein kinase activation. It can also increase intracellular calcium levels and stimulate vascular endothelial growth factors, thereby increasing production of nitric oxide. Moreover, hydrogen functions to open the potassium channel sensitive to adenosine triphosphate and activate downstream mitogen-activated protein kinase pathways, thus promoting angiogenesis (61). Further experimental studies indicate that hydrogen prevents arterial intimal hyperplasia and atherosclerosis by inhibiting ROS and TNF-α/NF-κB pathways (62, 63); inhibits macrophage-derived foam cell apoptosis; stabilizes atherosclerotic plaques (64); reduces vascular stenosis; and promotes formation of vascular collaterals using the FIk1-Notch signal stimulated by paracrine VEGFs, thereby improving local microcirculation (61).

### Discussion on Hydrogen Therapy

There are multiple ways to introduce hydrogen into the body, including oral and intravenous injection of hydrogen water. However, since hydrogen has short half-life in the body and cannot be retained in the body's tissues for a long time, non-inhalation methods may not deliver satisfactory results. The therapeutic effects of this study group appeared at least 2 weeks after hydrogen inhalation in all the cases. From patients with long-term hydrogen inhalation, it has been observed that as hydrogen inhalation was continued for longer time, the patients' air- and bone-conduction hearing thresholds decreased more significantly. Fransson AE and his team also found repeated administration of H_2_ inhalation may further improve the therapeutic effect (65). After hydrogen inhalation was stopped, the patients' bone- and air-conduction hearing thresholds rose. Given that hearing loss in this study group lasted for months or even years, it is speculated that the disease had “aged”. The effect of hydrogen depends on sufficient dose and time. It seems that only inhalation of hydrogen can meet these requirements.

The concentration level and flow rate of inhaled hydrogen are also important. The hydrogen inhaled by this study group has a flow rate of 3L/min and concentration level at 67%. These are the maximum levels that can be provided by the only hydrogen inhalation equipment approved by the Chinese authority.

So far, there has been no evidence as to whether inhalation of pure hydrogen is better or inhalation of hydrogen-oxygen mixture is. If high-concentration pure hydrogen is to be inhaled, oxygen intake is inevitably affected. Inhaling a mixture of hydrogen and oxygen helps at least prevent hypoxia. Studies have revealed that during inhalation of hydrogen-oxygen mixture, hydrogen molecules, which are extremely small and permeable, can “carry” larger molecules of oxygen to the deep areas of tissues and improve oxygen supply (66). The mixture inhaled by this study group contains 33% oxygen, which exceeds the oxygen level in normal air.

## Conclusion

Hearing loss is a common and persisting adverse reaction for nasopharyngeal carcinoma patients with radiotherapy. The hearing loss of such patients is usually long-lasting, chronical and gradual, thus affecting the quality of life in a serious manner. Unfortunately, there has been no special treatment available so far. Hydrogen inhalation can improve the hearing of long-term surviving patients with nasopharyngeal carcinoma after radiotherapy, and achieves more significant efficacy as the duration of treatment is extended. Its mechanism of action might be associated with hydrogen's functions in controlling oxidative stress and inflammation; protecting cells; and improving blood perfusion. This study represents the first effort to adopt hydrogen inhalation as an independent measure for improving hearing loss in the patients with long-term survival of nasopharyngeal cancer. If this study would be supported by more experiments and verification in the future, hydrogen inhalation would be proven a safe and effective means of rehabilitation for the challenge in the medical world.

## Data Availability Statement

The raw data supporting the conclusions of this article will be made available by the authors, without undue reservation.

## Ethics Statement

The studies involving human participants were reviewed and approved by the Ethics Committee of Fuda Cancer Hospital, Jinan University, China. The patients/participants provided their written informed consent to participate in this study.

## Author Contributions

KX developed the idea for and was involved in the design of this study. XK and TL reviewed available data sources and drafted the manuscript. Y-YL, ZY, and KX critically revised the manuscript. All authors read and approved the final manuscript.

## Conflict of Interest

The authors declare that the research was conducted in the absence of any commercial or financial relationships that could be construed as a potential conflict of interest.

## Publisher's Note

All claims expressed in this article are solely those of the authors and do not necessarily represent those of their affiliated organizations, or those of the publisher, the editors and the reviewers. Any product that may be evaluated in this article, or claim that may be made by its manufacturer, is not guaranteed or endorsed by the publisher.
